# COVID-19 infection and severe clinical outcomes in patients with kidney disease by vaccination status: a nationwide cohort study in Korea

**DOI:** 10.4178/epih.e2024065

**Published:** 2024-07-17

**Authors:** Jieun Woo, Ahhyung Choi, Jaehun Jung, Ju-Young Shin

**Affiliations:** 1 Department of Biohealth Regulatory Science, Sungkyunkwan University, Suwon, Korea; 2 School of Pharmacy, Sungkyunkwan University, Suwon, Korea; 3 Harvard-MIT Center for Regulatory Science, Harvard Medical School, Boston, MA, USA; 4 Department of Preventive Medicine, Gachon University College of Medicine, Incheon, Korea; 5 Artificial Intelligence and Big-Data Convergence Center, Gil Medical Center, Gachon University College of Medicine, Incheon, Korea; 6 Samsung Advanced Institute for Health Sciences & Technology, Sungkyunkwan University, Seoul, Korea

**Keywords:** COVID-19, COVID-19 vaccine, Kidney disease, Pharmacoepidemiology

## Abstract

**OBJECTIVES:**

Patients with kidney disease have been prioritized for coronavirus disease 2019 (COVID-19) vaccination due to their susceptibility to COVID-19 infection. However, little evidence exists regarding these patients’ vulnerability to COVID-19 post-vaccination. Thus, we evaluated the risk of COVID-19 in patients with kidney disease compared to individuals without kidney disease according to vaccination status.

**METHODS:**

A retrospective cohort study was conducted using the Korean nationwide COVID-19 registry linked with National Health Insurance Service claims data (2018-2021). Among individuals aged 12 years or older, 2 separate cohorts were constructed: a COVID-19-vaccinated cohort and an unvaccinated cohort. Within each cohort, the risk of COVID-19 infection and all-cause mortality, hospitalization, and emergency room visits within 30 days of COVID-19 infection were compared between patients with and without kidney disease. To adjust for potential confounding, we used propensity score matching. Hazard ratios (HRs) for each outcome were estimated using a Cox proportional hazard model.

**RESULTS:**

We identified 785,390 and 836,490 individuals in the vaccinated and unvaccinated cohorts, respectively. Compared to patients without kidney disease, patients with kidney disease were at a higher risk of COVID-19 infection in both the vaccinated cohort (HR, 1.08; 95% confidence interval [CI], 1.02 to 1.16) and the unvaccinated cohort (HR, 1.09; 95% CI, 0.99 to 1.20). Likewise, patients with kidney disease generally were at higher risk for severe clinical outcomes within 30 days of COVID-19 infection. Subgroup and sensitivity analyses showed generally consistent results.

**CONCLUSIONS:**

Our study observed excess risk of COVID-19 in patients with kidney disease, highlighting the importance of ongoing attention to these patients even post-vaccination.

## INTRODUCTION

Coronavirus disease 2019 (COVID-19) has become an ongoing pandemic, infecting over 768 million people and causing approximately 7 million deaths worldwide [1]. A variety of strategies have been proposed to mitigate the impact of COVID-19 [2-6]. Among them, vaccines are considered to be one of the most effective approaches, demonstrating a reduced risk of COVID-19 infection and associated hospitalization or mortality [7-9]. As a result, COVID-19 vaccinations have been widely recommended, particularly for individuals with chronic comorbidities such as chronic kidney disease [10-12], as these populations are considered more susceptible to COVID-19 [13,14].

Patients with chronic kidney disease are recognized as a high-risk group for COVID-19, primarily due to their compromised immune function [15]. However, it remains uncertain whether individuals with kidney disease continue to be disproportionately vulnerable to COVID-19 after vaccination. One study has reported a higher mortality rate among patients undergoing hemodialysis than among the general population during the Omicron BA.5 dominant period; however, whether these individuals had been vaccinated was unknown [16]. In another study, immune response following COVID-19 vaccination in patients with advanced stages of chronic kidney disease was reported to be comparable to that in controls [17]. Nonetheless, there still remains a knowledge gap in real-world evidence on the risk of COVID-19 infection and its prognosis among patients with kidney disease, including those in the early stages.

Therefore, we aimed to evaluate the risk of COVID-19 infection, mortality, hospitalization, and emergency room visits after COVID-19 infection in patients with kidney disease compared to those without kidney disease based on vaccination status.

## MATERIALS AND METHODS

### Data source and study design

We conducted a retrospective cohort study using the Korea Disease Control and Prevention Agency-COVID-19-National Health Insurance Service cohort (K-COV-N cohort) between July 2018 and December 2021. The K-COV-N cohort was constructed by linking the National Health Information Database (NHID), the COVID-19 infection registry, and the COVID-19 vaccination registry in Korea.

The NHID represents the entire population in Korea (approximately 50 million) and includes information on socio-demographic characteristics (e.g., age, sex, and health insurance type), the date of death based on linkage to the national vital statistics, and medical records of reimbursement from all settings (e.g., inpatient, outpatient, and emergency room visits). In the NHID, diagnoses are recorded based on the International Classification of Diseases 10th revision (ICD-10), and prescriptions are recorded with the National Drug Codes, which are mapped to the Anatomical Therapeutic Chemical classification.

The COVID-19 infection and vaccination registry is operated by the Korea Disease Control and Prevention Agency (KDCA) and includes individual-level data on all polymerase chain reaction (PCR)-confirmed COVID-19 infections and vaccination information including vaccination date, type, and frequency. In Korea, the COVID-19 vaccine has been available since February 26, 2021. Due to the NHID provision policy, we used 25% random sampling of the data source stratified by age and sex.

### Study population and outcomes of interests

For the vaccinated cohort, we identified all vaccinated individuals aged 12 years or older between February 26, 2021 and November 30, 2021. We then excluded individuals with COVID-19 infection before vaccination and those who had a kidney transplant pre-vaccination. Subsequently, the cohort was divided into patients with and without kidney disease, with kidney disease defined as having at least 1 inpatient or 2 outpatient diagnoses recorded before vaccination (Supplementary Material 1).

The primary outcome was COVID-19 infection, and the secondary outcomes were all-cause mortality, hospitalization, and emergency room visits within 30 days of COVID-19 infection. In the vaccinated cohort, individuals were followed from the vaccination date until the date of outcome occurrence, death, or end of the study period (December 31, 2021), whichever came first.

We also evaluated the risk of study outcomes among the unvaccinated population aged 12 years or older during the study period. Similar to the vaccinated cohort, we compared the risk of study outcomes in patients with and without kidney disease in this unvaccinated cohort. The follow-up started on February 26, 2021, the time when COVID-19 vaccination started in Korea, and ended on the outcome occurrence, vaccination, death, or end of the study period (December 31, 2021), whichever came first.

### Covariates

We considered several potential confounders: demographic characteristics such as age, sex, and income level, and comorbidities such as diabetes, hyperlipidemia, hypertension, cancer, liver disease and chronic lung disease, and the Charlson comorbidity index (CCI) [18]. In the vaccination cohort, the type of COVID-19 vaccine (e.g., BNT162b2, mRNA-1273, ChAdOx1-S, Ad.26. COV2.S) was additionally considered. Demographic characteristics were measured at the date of COVID-19 vaccination for the vaccinated cohort and on February 26, 2021 for the unvaccinated cohort, and comorbidities and CCI were measured during the past 1 year before COVID-19 vaccination or February 26, 2021 for the vaccinated cohort and unvaccinated cohort, respectively. Detailed definitions of the comorbidities are available in Supplementary Material 1.

### Statistical analysis

The baseline characteristics of the study populations are presented as mean and standard deviation (SD) for continuous variables, and as frequencies with percentages for categorical variables. To assess covariate balance, we calculated absolute standardized differences (aSD), considering aSD> 0.1 as imbalanced.

The propensity score (PS) was calculated using multivariable logistic regression, with kidney disease as the dependent variable and all the predefined covariates (e.g., demographic characteristics, comorbidities, and CCI) as independent variables [19]. To adjust for potential confounding, patients with kidney disease were 1:4 matched to those without based on their estimated PS by applying a nearest-neighbor-matching algorithm with a caliper width of 0.05 [20]. In the PS-matched cohort, we calculated the incidence rates per 1,000 person-years, and the hazard ratios (HRs) with 95% confidence intervals (CIs) using the Cox proportional hazard regression model while adjusting for covariates that were imbalanced even after PS matching.

All statistical analyses were performed with SAS Enterprise Guide version 7.1 (SAS Institute Inc., Cary, NC, USA), and a 2-sided α value less than 0.05 was considered statistically significant.

### Subgroup and sensitivity analyses

We conducted several subgroup analyses, stratified by dialysis status, age (12-17, 18-44, 45-64, or ≥ 65 years), sex (male or female), CCI (0, 1, 2, or ≥ 3), and vaccination dose (partial or full). Vaccination was defined as partial if 1 dose of the BNT162b2, mRNA-1273, or ChAdOx1-S vaccine was received prior to COVID-19 infection, and considered full if 2 doses of the BNT162b2, mRNA-1273, or ChAdOx1-S vaccine or 1 dose of the Ad.26. COV2.S vaccine was received prior to COVID-19 infection. Additionally, as a sensitivity analysis, we redefined the secondary outcomes as all-cause mortality, hospitalization, and emergency room visits within 14 days after COVID-19 infection instead of 30 days in our main analysis. We narrowed the evaluation period based on the assumption that outcomes occurring within this closer timeframe to COVID-19 infection would be more likely to be associated with COVID-19 infection.

### Ethics statement

Ethical approval was obtained from the Institutional Review Board of Sungkyunkwan University, where requirement of informed consent was waived as this study used anonymized administrative data (IRB No. SKKU 2023-04-012).

## RESULTS

### Characteristics of patients with and without kidney disease

**Figure 1.**
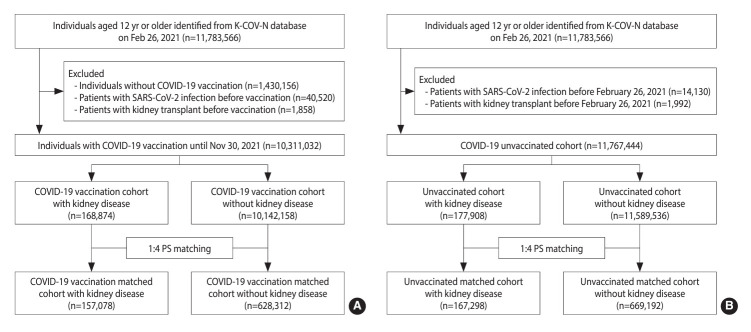
Flow chart of patient selection in (A) the vaccinated cohort and (B) the unvaccinated cohort. K-COV-N, Korea Disease Control and Prevention Agency-COVID-19-National Health Insurance Service; COVID-19, coronavirus disease 2019; SARS-CoV-2, severe acute respiratory syndrome coronavirus 2; PS, propensity score.

In the vaccinated cohort, after applying the exclusion criteria, we identified 10,311,032 individuals, of whom 168,874 were patients with kidney disease and 10,142,158 were patients without kidney disease. Before PS matching, compared to the patients without kidney disease, patients with kidney disease were older and more likely to have comorbidities (Supplementary Material 2). After 1:4 PS matching, 785,390 vaccinated patients were included, with 157,078 having kidney disease and 628,312 without kidney disease (Figure 1A). All baseline covariates were well balanced between these 2 groups (aSD<0.1) (Table 1). In the unvaccinated cohort, we identified 836,490 matched patients, of whom 167,298 were patients with kidney disease and 669,192 were patients without kidney disease (Figure 1B). After 1:4 PS matching, all the baseline covariates except chronic lung disease were well balanced between the 2 groups (Table 1).

### Risk of coronavirus disease 2019 in patients with and without kidney disease

In the PS-matched vaccinated cohort, the incidence rate of COVID-19 infection was higher in patients with kidney disease (14.5 per 1,000 person-years) than in patients without kidney disease (13.5 per 1,000 person-years). Patients with kidney disease had higher risks of COVID-19 infection (HR, 1.08; 95% CI, 1.02 to 1.16), all-cause mortality (HR, 1.89; 95% CI, 1.38 to 2.60), hospitalization (HR, 1.17; 95% CI, 1.07 to 1.29), and emergency room visits (HR, 1.25; 95% CI, 1.02 to 1.53) after COVID-19 infection. Likewise, in the unvaccinated cohort, the risks of COVID-19 were generally higher in patients with kidney disease than in those without, although there was no significant difference in hospitalization (Table 2). The incidence rates of COVID-19 infection were slightly higher in patients with kidney disease in both the vaccinated and unvaccinated cohorts (Supplementary Materials 3 and 4).

### Subgroup and sensitivity analyses

Subgroup analyses based on dialysis status are shown in Table 3. Consistent with our main findings, vaccinated patients with kidney disease who were not on dialysis were at increased risk of COVID-19. However, among patients with kidney disease who were on dialysis, although a higher risk of all-cause mortality after the COVID-19 infection was observed (HR, 2.48; 95% CI, 1.27 to 4.86), no significant differences were found in the risk of COVID-19 infection, hospitalization, and emergency room visits after the COVID-19 infection (Table 3). Similarly, in the unvaccinated cohort, the risk of COVID-19 was mostly higher in patients with kidney disease regardless of dialysis status, except for non-differential risk observed for hospitalization after COVID-19 infection in the non-dialysis group (Table 3).

The results of subgroup analyses stratified by age, sex, CCI, and vaccination dose were generally consistent with the main findings (Supplementary Materials 5 and 6). The sensitivity analysis, in which we redefined the follow-up period of secondary outcomes as 14 days within COVID-19 infection, also presented similar results (Table 4).

## DISCUSSION

In this large nationwide cohort study, we found that patients with kidney disease were at higher risk of COVID-19 infection, all-cause mortality, hospitalization, and emergency room visits after COVID-19 infection than patients without kidney disease, even after vaccination. These results were generally consistent across the various subgroup and sensitivity analyses, and similar results were observed in the unvaccinated population. Overall, our findings have shown that patients with kidney disease are vulnerable to COVID-19, regardless of the vaccination status, indicating the importance of ongoing care for these populations even after vaccination.

Several previous studies have demonstrated the excess risk of COVID-19 in patients with kidney disease compared to patients without kidney disease. According to a meta-analysis that assessed the risk of various COVID-19-related outcomes, patients with chronic kidney disease were associated with a higher risk of mortality than patients without kidney disease (HR, 1.48, 95% CI, 1.33 to 1.65) [21]. In another cohort study, compared to patients without kidney disease, patients with reduced kidney function showed a higher risk of COVID-19-related death, with results presented based on the severity of kidney damage (stage 3-4: HR, 1.33; 95% CI, 1.28 to 1.40; stage 5: HR, 2.52; 95% CI, 2.33 to 2.72) [22]. However, all these studies were conducted prior to the approval of COVID-19 vaccines [23]; thus, the study populations were those who did not receive COVID-19 vaccination. Therefore, a knowledge gap existed regarding whether patients with kidney disease remained a vulnerable group after vaccination. Post-vaccination immune response studies reported that anti-spike antibody development after the COVID-19 vaccine approached 100% in patients with chronic kidney disease, as in the patients without kidney disease [17,24,25]. However, even with high seroconversion, real-world COVID-19 infection rates and its prognosis had yet to be evaluated. Our study, based on large nationwide representative real-world data, found that vaccinated patients with kidney disease present a higher risk of COVID-19, even after the vaccination.

There may be several biologically plausible mechanisms that link kidney disease to the risk of COVID-19 infection. Patients with kidney disease have a compromised immune system with decreased activity of natural killer cells and an imbalanced ratio of CD4+/CD8+ T cells, which may lead to susceptibility to COVID-19 infection [2]. In addition, angiotensin-converting enzyme-2 (ACE2), which is highly expressed in kidney epithelial cells and bladder cells [26,27], was identified as a functional receptor for severe acute respiratory syndrome coronavirus-2 (SARS-CoV-2) [28,29]. SARS-CoV-2, the causative agent of COVID-19 [30], could bind to kidney cells during infection and cause kidney damage, resulting in acute tubular necrosis, glomerulopathy, and protein leakage from Bowman’s capsule [31], all of which may lead to a poor prognosis after the COVID-19 infection.

Our study has several key strengths. First, to our knowledge, this is the first observational study that examined the risk of COVID-19 after vaccination in patients with kidney disease compared to patients without kidney disease. Our study included patients with kidney disease regardless of dialysis status, and the results stratified by dialysis status were demonstrated through a subgroup analysis. Moreover, by using a large, nationwide database, we were able to include more than 150,000 patients with kidney disease and derive relatively precise estimates. Lastly, as the KDCA mandatorily collects all the COVID-19 infection cases in Korea and our data set obtained the COVID-19 infection records from the KDCA, our definition of COVID-19 has high sensitivity.

This study also has several limitations. First, although we adjusted for various measurable covariates by applying PS matching and multivariate adjustment, unmeasured or residual confounding factors may exist. Second, our secondary outcomes, which were all-cause mortality, hospitalization, and emergency room visits within 30 days of COVID-19 infection, may not be the direct consequences of COVID-19 infection, as we were unable to identify its cause in our NHID database. However, sensitivity analyses where we redefined the assessment period as 14 days to better address the prognosis showed consistent results. Third, our database did not contain laboratory data such as the estimated glomerular filtration rate (eGFR), which limited our ability to define chronic kidney disease or acute renal failure based on clinical manifestations. Although we defined kidney disease with reference to previous studies using diagnostic codes, further studies using laboratory data, including the eGFR, may be informative. Fourth, our study period was until December 2021, the time period when the Alpha and Delta variants were predominant in Korea [32]. Given that the Omicron variant differs from the Alpha or Beta variants in several characteristics, further studies including the period of Omicron variant dominance are needed. Fifth, mild or asymptomatic cases of COVID-19 infection may not have been captured in our database. However, during our study period, Korea had a strict policy on COVID-19. Regardless of symptoms, all patients suspected of COVID-19 who had close contact with COVID-19 patients were obligatorily tested by PCR [33,34]. Thus, we were able to capture numerous asymptomatic cases; however, there still exists a possibility of missing some cases.

In conclusion, the risk of COVID-19 infection and all-cause mortality, hospitalization, and emergency room visits after COVID-19 infection was higher in patients with kidney disease than in patients without kidney disease. Therefore, patients with kidney disease should be continuously monitored for COVID-19 even after vaccination and remain cautious. By identifying susceptible populations within a largely vaccinated and unvaccinated population, our study expands the available evidence and further suggests the need to establish risk-mitigation strategies for patients with kidney disease.

## Figures and Tables

**Table 1.** Baseline characteristics of the patients with and without kidney disease after PS matching

Characteristics	Vaccinated cohort			Unvaccinated cohort		
	Patients with kidney disease (n=157,078)	Patients without kidney disease (n=628,312)	aSD	Patients with kidney disease (n=167,298)	Patients without kidney disease (n=669,192)	aSD
Age (yr)			0.00			0.04
Mean±SD	65.2±14.3	65.7±14.2	0.04	65.6±14.7	66.2±14.4	0.04
12-17	245 (0.2)	888 (0.1)		467 (0.3)	1,654 (0.2)	
18-44	12,982 (8.3)	47,330 (7.5)		13,883 (8.3)	49,634 (7.4)	
45-64	57,248 (36.4)	226,587 (36.1)		58,874 (35.2)	232,285 (34.7)	
≥65	86,603 (55.1)	353,507 (56.3)		94,074 (56.2)	385,619 (57.6)	
Sex			0.07			0.07
Male	90,934 (57.9)	340,716 (54.2)		96,099 (57.4)	360,395 (53.9)	
Female	66,144 (42.1)	287,596 (45.8)		71,199 (42.6)	308,797 (46.1)	
Income level			0.03			0.03
1st quartile	42,077 (26.8)	166,038 (26.4)		46,214 (27.6)	177,755 (26.6)	
2nd quartile	26,048 (16.6)	107,298 (17.1)		27,521 (16.5)	114,438 (17.1)	
3rd quartile	32,736 (20.8)	134,578 (21.4)		34,390 (20.6)	142,295 (21.3)	
4th quartile	56,217 (35.8)	220,398 (35.1)		59,173 (35.4)	234,704 (35.1)	
Comorbidities						
Anemia	14,638 (9.3)	57,293 (9.1)	0.01	17,034 (10.2)	58,618 (8.8)	0.05
Cancer	28,249 (18.0)	119,432 (19.0)	0.03	29,112 (17.4)	122,977 (18.4)	0.03
Cardiac dysrhythmias	9,520 (6.1)	46,085 (7.3)	0.05	10,391 (6.2)	49,361 (7.4)	0.05
Chronic lung disease	21,417 (13.6)	105,381 (16.8)	0.09	24,174 (14.4)	123,009 (18.4)	0.11
Congestive heart failure	16,611 (10.6)	71,500 (11.4)	0.03	18,410 (11.0)	81,944 (12.2)	0.04
Coronary artery disease	18,181 (11.6)	78,510 (12.5)	0.03	19,742 (11.8)	86,081 (12.9)	0.03
Dementia	13,304 (8.5)	61,996 (9.9)	0.05	14,711 (8.8)	67,335 (10.1)	0.04
Depression	9,300 (5.9)	48,585 (7.7)	0.07	10,174 (6.1)	47,354 (7.1)	0.04
Diabetes	87,175 (55.5)	360,930 (57.4)	0.04	92,167 (55.1)	373,500 (55.8)	0.02
Hyperlipidemia	61,759 (39.3)	265,883 (42.3)	0.06	62,726 (37.5)	264,881 (39.6)	0.04
Hypertension	86,782 (55.2)	345,739 (55.0)	0.00	91,478 (54.7)	372,064 (55.6)	0.02
Hypothyroidism	6,128 (3.9)	29,826 (4.7)	0.04	6,328 (3.8)	29,977 (4.5)	0.04
Liver disease	16,315 (10.4)	79,340 (12.6)	0.07	16,814 (10.1)	85,411 (12.8)	0.09
Peripheral vascular disease	11,771 (7.5)	57,811 (9.2)	0.06	12,506 (7.5)	55,323 (8.3)	0.03
Stroke	12,204 (7.8)	58,373 (9.3)	0.05	13,761 (8.2)	58,584 (8.8)	0.02
CCI, mean±SD	1.4±1.1	1.4±1.4	0.01	1.4±1.1	1.4±1.4	0.04
First vaccine type			0.08			-
ChAdOx1-S	69,181 (44.0)	277,720 (44.2)		N/A	N/A	
BNT162b2	73,729 (46.9)	287,595 (45.8)		N/A	N/A	
mRNA-1273	11,987 (7.6)	52,631 (8.4)		N/A	N/A	
Ad.26.COV2.S	1,973 (1.3)	9,191 (1.5)		N/A	N/A	
Second vaccine type			0.08			-
ChAdOx1-S	58,613 (37.3)	243,651 (38.8)		N/A	N/A	
BNT162b2	81,661 (52.0)	309,554 (49.3)		N/A	N/A	
mRNA-1273	11,480 (7.3)	48,807 (7.8)		N/A	N/A	

Values are presented as number (%). PS, propensity score; aSD, absolute standardized difference; SD, standard deviation; CCI, Charlson comorbidity index; N/A, not available.

**Table 2.** COVID-19 infection and severe clinical outcomes in patients with and without kidney disease

Variables	Vaccinated cohort		Unvaccinated cohort^1^	
	Patients with kidney disease (n=157,078)	Patients without kidney disease (n=628,312)	Patients with kidney disease (n=167,298)	Patients without kidney disease (n=669,192)
COVID-19 infection				
No. of events	1,202	4,464	523	1,782
PY	82,618	331,619	60,112	238,098
IR per 1,000 PY	14.5	13.5	8.7	7.5
aHR (95% CI)	1.08 (1.02, 1.16)	1.00 (reference)	1.09 (0.99, 1.20)	1.00 (reference)
All-cause mortality				
No. of events	56	119	52	79
PY	82,693	331,905	60,146	238,222
IR per 1,000 PY	0.7	0.4	0.9	0.3
aHR (95% CI)	1.89 (1.38, 2.60)	1.00 (reference)	2.30 (1.62, 3.26)	1.00 (reference)
Hospitalization				
No. of events	578	1,988	249	914
PY	82,658	331,779	60,131	238,163
IR per 1,000 PY	7.0	6.0	4.1	3.8
aHR (95% CI)	1.17 (1.07, 1.29)	1.00 (reference)	1.00 (0.87, 1.15)	1.00 (reference)
Emergency room visits				
No. of events	123	396	69	194
PY	82,688	331,883	60,143	238,212
IR per 1,000 PY	1.5	1.2	1.1	0.8
aHR (95% CI)	1.25 (1.02, 1.53)	1.00 (reference)	1.30 (0.99, 1.72)	1.00 (reference)

COVID-19, coronavirus disease 2019; PY, person-years; IR, incidence rate; aHR, adjusted hazard ratio; CI, confidence interval.

1 Covariates with imbalance after propensity score matching (chronic lung disease) were adjusted in unvaccinated cohort.

**Table 3.** Subgroup analyses on the risk of COVID-19 by dialysis status

Variables	Vaccinated cohort				Unvaccinated cohort			
	Non-dialysis patients with kidney disease (n=147,776)	Patients without kidney disease (n=591,104)	Dialysis patients with kidney disease (n=13,635)^1^	Patients without kidney disease (n=54,540)	Non-dialysis patients with kidney disease (n=154,234)	Patients without kidney disease (n=616,936)	Dialysis patients with kidney disease (n=18,278)^2^	Patients without kidney disease (n=73,112)
COVID-19 infection								
No. of events	1,152	4,162	85	429	478	1,633	68	183
PY	77,184.2	313,379.6	7,727.3	30,879.0	56,172.9	219,129.5	5,656.7	26,160.7
IR per 1,000 PY	14.9	13.3	11.0	13.9	8.5	7.5	12.0	7.0
aHR (95% CI)	1.14 (1.07, 1.22)	1.00 (reference)	0.77 (0.61, 0.98)	1.00 (reference)	1.08 (0.98, 1.20)	1.00 (reference)	1.09 (0.99, 1.20)	1.00 (reference)
All-cause mortality								
No. of events	48	116	13	29	49	72	14	17
PY	77,256.3	313,647.1	7,731.9	30,906.2	56,204.2	219,243.1	5,660.6	26,172.9
IR per 1,000 PY	0.6	0.4	1.7	0.9	0.9	0.3	2.5	0.6
aHR (95% CI)	1.75 (1.25, 2.45)	1.00 (reference)	2.48 (1.27, 4.86)	1.00 (reference)	2.37 (1.65, 3.40)	1.00 (reference)	3.73 (1.81, 7.69)	1.00 (reference)
Hospitalization								
No. of events	550	1905	48	213	220	826	40	101
PY	77,223.0	313,527.2	7,729.3	30,893.4	56,190.5	219,189.7	5,658.7	26,166.8
IR per 1,000 PY	7.1	6.1	6.2	6.9	3.9	3.8	7.1	3.9
aHR (95% CI)	1.20 (1.09, 1.31)	1.00 (reference)	0.90 (0.66, 1.24)	1.00 (reference)	0.98 (0.84, 1.14)	1.00 (reference)	1.53 (1.06, 2.21)	1.00 (reference)
Emergency room visits								
No. of events	118	372	11	45	60	186	11	13
PY	77,250.6	313,626.1	7,731.6	30,903.9	56,201.0	219,232.4	5,660.2	26,172.1
IR per 1,000 PY	1.5	1.2	1.4	1.5	1.1	0.8	1.9	0.5
aHR (95% CI)	1.32 (1.07, 1.62)	1.00 (reference)	0.96 (0.49, 1.87)	1.00 (reference)	1.18 (0.88, 1.58)	1.00 (reference)	3.24 (1.44, 7.30)	1.00 (reference)

COVID-19, coronavirus disease 2019; PY, person-years; IR, incidence rate; aHR, adjusted hazard ratio; CI, confidence interval.

1 Covariates with imbalances after propensity score matching (age, chronic lung disease, dementia, diabetes, hyperlipidemia, liver disease, peripheral vascular disease) were adjusted.

2 Covariates with imbalances after propensity score matching (age, anemia, cardiac dysrhythmias, chronic lung disease, depression, diabetes) were adjusted.

**Table 4.** Sensitivity analysis of the risk of COVID-19 by redefining the follow-up period as 14 days within COVID-19 infection

Variables	Vaccinated cohort		Unvaccinated cohort	
	Patients with kidney disease (n=157,078)	Patient without kidney disease (n=628,312)	Patients with kidney disease (n=167,298)^1^	Patient without kidney disease (n=669,192)
All-cause mortality				
No. of events	36	64	42	52
PY	82,660.2	331,776.3	60,130.1	238,162.4
IR per 1,000 PY	0.4	0.2	0.7	0.2
aHR (95% CI)	2.26 (1.50, 3.40)	1.00 (reference)	2.80 (1.86, 4.20)	1.00 (reference)
Hospitalization				
No. of events	555	1,912	231	868
PY	82,642.6	331,713.7	60,122.9	238,133.9
IR per 1,000 PY	6.7	5.8	3.8	3.6
aHR (95% CI)	1.17 (1.06, 1.29)	1.00 (reference)	0.98 (0.84, 1.13)	1.00 (reference)
Emergency room visits				
No. of events	102	361	57	158
PY	82,657.4	331,765.4	60,128.5	238,157.5
IR per 1,000 PY	1.2	1.1	0.9	0.7
aHR (95% CI)	1.14 (0.91, 1.42)	1.00 (reference)	1.31 (0.97, 1.78)	1.00 (reference)

COVID-19, coronavirus disease 2019; PY, person-years; IR, incidence rate; aHR, adjusted hazard ratio; CI, confidence interval.

1 Covariates with imbalances after propensity score matching (chronic lung disease) were adjusted.

## Supplementary materials

Supplementary Material 1. Definitions and codes for variables identified in the study

Supplementary Material 2. Baseline characteristics of the patients with and without kidney disease before PS- matching

Supplementary Material 3. Time trend of incidence proportions of COVID-19 infection in vaccinated cohort

Supplementary Material 4. Time trend of incidence proportions of COVID-19 infection in unvaccinated cohort

Supplementary Material 5. Subgroup analysis on the risk of COVID-19 and severe clinical outcomes in patients with kidney disease compared to patients without kidney disease in vaccinated cohort

Supplementary Material 6. Subgroup analysis on the risk of COVID-19 and severe clinical outcomes in patients with kidney disease compared to patients without kidney disease in unvaccinated cohort
