# Supplementary material for: COVID-19 infection and severe clinical outcomes in patients with kidney disease by vaccination status: a nationwide cohort study in Korea
Source: Epidemiol Health. 2024 Jul 17;46:e2024065. doi: 10.4178/epih.e2024065 (PMC11576527; doi:10.4178/epih.e2024065)
Supplement: Supplementary Material 1. — Definitions and codes for variables identified in the study [file epih-46-e2024065-Supplementary-1.docx]

**Supplementary Material 1. Definitions and codes for variables identified in the study**

| **Disease status** | **Type of code** | **Codes** |
| --- | --- | --- |
| Chronic kidney disease^1-3^ | ICD-10 code | N03, N05, N165, N18, N19, N250, I12, I13, I15.0, I15.1, Z490, Z491, Z492, Z940, Z992, E102, E112, E132, E142, T861 |
| Dialysis | Procedural code | O701, O702, O703, O704, O705, O706, O707, O708 |
| Anemia | ICD-10 code | D460, D461, D462, D464, D465, D466, D50-53, D55-64, O990, P612, P613, P614 |
| Atherosclerotic coronary artery disease | ICD-10 code | I250, I251, I70 |
| Cancer | ICD-10 code | C00-97, D00-48 |
| Cardiac dysrhythmias | ICD-10 code | I44-49, R00 |
| Chronic lung disease | ICD-10 code | J12-18, J40-46, J60-67, J684, J69, J701, J703, J841, J920, J961, J982, J983, O290, O740, O890, P230, P278, P350 |
| Congestive heart failure | ICD-10 code | I110, I130, I132, I42, I50 |
| Dementia | ICD-10 code | F00, F01, F02, F03, F051, G30, G31, R54 |
| Diabetes | ICD-10 code | E10, E11, E12, E13, E14, O240, O241, O242, O243 |
| Hyperlipidemia | ICD-10 code | E780, E781, E782, E783, E784, E785 |
| Hypertension | ICD-10 code | I10, I11, I12, I13, I14, I15, I270, I272 |
| Liver disease | ICD-10 code | K70, K713, K714, K715, K717, K721, K729, K73, K74, K76 |
| Peripheral vascular disease | ICD-10 code | I73, I74, I77, I78, I79, R02, Z958, Z959 |
| Stroke | ICD-10 code | I60, I61, I62, I63, I64, I69, G45 |

**Abbreviation**: ICD-10 code=International Classification of Diseases 10^th^ code

^1^Kristensen JH, Basit S, Wohlfahrt J, Damholt MB, Boyd HA. Pre-eclampsia and risk of later kidney disease: nationwide cohort study. *Bmj* 2019; **365**: l1516.

^2^Kang SH, Kim SW, Kim AY, Cho KH, Park JW, Do JY. Association between Chronic Kidney Disease or Acute Kidney Injury and Clinical Outcomes in COVID-19 Patients. *J Korean Med Sci* 2020; **35**(50): e434.

^3^Kim SH, Jo MW, Go DS, Ryu DR, Park J. Economic burden of chronic kidney disease in Korea using national sample cohort. *J Nephrol* 2017; **30**(6): 787-93.
