# Supplementary material for: COVID-19 infection and severe clinical outcomes in patients with kidney disease by vaccination status: a nationwide cohort study in Korea
Source: Epidemiol Health. 2024 Jul 17;46:e2024065. doi: 10.4178/epih.e2024065 (PMC11576527; doi:10.4178/epih.e2024065)
Supplement: Supplementary Material 2. — Baseline characteristics of the patients with and without kidney disease before PS- matching [file epih-46-e2024065-Supplementary-2.docx]

**Supplementary Material 2. Baseline characteristics of the patients with and without kidney disease** **before PS- matching**

|  | | **Vaccinated cohort** | |  |  | **Unvaccinated cohort** | |  |
| --- | --- | --- | --- | --- | --- | --- | --- | --- |
|  |  | **Patients with**  **Kidney disease** | **Patients without**  **Kidney disease** | **aSD** |  | **Patients with**  **Kidney disease** | **Patients without**  **Kidney disease** | **aSD** |
|  |  | **(n=168,874)** | **(n=10,142,158)** |  |  | **(n=177,908)** | **(n=11,589,536)** |  |
| **Age, mean(SD)** | | 65.8 (14.3) | 48.0 (18.2) | 1.09 |  | 66.2 (14.6) | 46.8 (18.9) | 1.15 |
| **Age group, n(%)** | |  |  | 1.05 |  |  |  | 1.13 |
|  | 12-17 | 245 (0.1) | 319 217 (3.1) |  |  | 467 (0.3) | 681,838 (5.9) |  |
|  | 18-44 | 13,128 (7.8) | 4,006,635 (39.5) |  |  | 13,940 (7.8) | 4,607,181 (39.8) |  |
|  | 45-64 | 59,648 (35.3) | 3,846,046 (37.9) |  |  | 60,523 (34) | 4,145,514 (35.8) |  |
|  | ≥65 | 95,853 (56.8) | 1,970,260 (19.4) |  |  | 102,978 (57.9) | 2,155,003 (18.6) |  |
| **Sex, n(%)** | |  |  | 0.19 |  |  |  | 0.18 |
|  | Male | 99,179 (58.7) | 4,993,133 (49.2) |  |  | 103,328 (58.1) | 5,715,466 (49.3) |  |
|  | Female | 69,695 (41.3) | 5,149,025 (50.8) |  |  | 74,580 (41.9) | 5,874,070 (50.7) |  |
| **Income level, n(%)** | |  |  | 0.17 |  |  |  | 0.19 |
|  | 1st quartile | 45,793 (27.1) | 2,365,347 (23.3) |  |  | 49,592 (27.9) | 2,725,157 (23.5) |  |
|  | 2nd quartile | 27,756 (16.4) | 2,110,371 (20.8) |  |  | 28,988 (16.3) | 2,400,166 (20.7) |  |
|  | 3rd quartile | 34,823 (20.6) | 2,413,681 (23.8) |  |  | 36,291 (20.4) | 2,759,236 (23.8) |  |
|  | 4th quartile | 60,502 (35.8) | 3,252,759 (32.1) |  |  | 63,037 (35.4) | 3,704,977 (32.0) |  |
| **Comorbidities, n(%)** | |  |  |  |  |  |  |  |
|  | Anemia | 21,283 (12.6) | 244,007 (2.4) | 0.39 |  | 23,540 (13.2) | 266,465 (2.3) | 0.42 |
|  | Cancer | 31,311 (18.5) | 1,084,366 (10.7) | 0.22 |  | 31,904 (17.9) | 1,119,338 (9.7) | 0.24 |
|  | Cardiac dysrhythmias | 11,193 (6.6) | 224,239 (2.2) | 0.22 |  | 12,018 (6.8) | 237,470 (2.0) | 0.23 |
|  | Chronic lung disease | 23,729 (14.1) | 698,824 (6.9) | 0.24 |  | 26,545 (14.9) | 812,399 (7.0) | 0.26 |
|  | Congestive heart failure | 21,127 (12.5) | 180,152 (1.8) | 0.43 |  | 22,969 (12.9) | 194,405 (1.7) | 0.44 |
|  | Coronary artery disease | 22,012 (13.0) | 295,680 (2.9) | 0.38 |  | 23,528 (13.2) | 314,667 (2.7) | 0.40 |
|  | Dementia | 14,869 (8.8) | 271,592 (2.7) | 0.27 |  | 16,337 (9.2) | 295,728 (2.6) | 0.29 |
|  | Depression | 10,306 (6.1) | 362,728 (3.6) | 0.12 |  | 11156 (6.3) | 391,356 (3.4) | 0.14 |
|  | Diabetes | 98,057 (58.1) | 1,033,695 (10.2) | 1.17 |  | 101,986 (57.3) | 1,083,467 (9.3) | 1.18 |
|  | Hyperlipidemia | 67,046 (39.7) | 1,834,523 (18.1) | 0.49 |  | 67,588 (38.0) | 1,839,728 (15.9) | 0.52 |
|  | Hypertension | 95,735 (56.7) | 2,052,848 (20.2) | 0.81 |  | 99,725 (56.1) | 2,140,462 (18.5) | 0.84 |
|  | Hypothyroidism: | 7,022 (4.2) | 211,623 (2.1) | 0.12 |  | 7,193 (4.0) | 226,833 (2.0) | 0.12 |
|  | Liver disease | 17,507 (10.4) | 592,257 (5.8) | 0.17 |  | 17,924 (10.1) | 611,985 (5.3) | 0.18 |
|  | Peripheral vascular disease | 13,603 (8.1) | 303,491 (3.0) | 0.22 |  | 14,132 (7.9) | 315,985 (2.7) | 0.23 |
|  | Stroke | 14,338 (8.5) | 216,735 (2.1) | 0.29 |  | 15,831 (8.9) | 242,325 (2.1) | 0.30 |
| **CCI, mean(SD)** | | 1.5 (1.2) | 0.4 (0.8) | 1.07 |  | 1.5 (1.2) | 0.3 (0.8) | 1.10 |
| **First vaccine type** | |  |  | 0.43 |  |  |  | - |
|  | ChAdOx1-S | 74,673 (44.2) | 2,669,455 (26.3) |  |  | N/A | N/A |  |
|  | BNT162b2 | 79,689 (47.2) | 5,545,216 (54.7) |  |  | N/A | N/A |  |
|  | mRNA-1273 | 12,262 (7.3) | 1,599,599 (15.8) |  |  | N/A | N/A |  |
|  | Ad.26.COV2.S | 2,032 (1.2) | 314,274 (3.1) |  |  | N/A | N/A |  |
| **Second vaccine type** | |  |  | 0.43 |  |  |  | - |
|  | ChAdOx1-S | 61,468 (36.4) | 2,223,764 (21.9) |  |  | N/A | N/A |  |
|  | BNT162b2 | 89,852 (53.2) | 5,695,909 (56.2) |  |  | N/A | N/A |  |
|  | mRNA-1273 | 11,728 (6.9) | 1,524,538 (15.0) |  |  | N/A | N/A |  |

**Abbreviation**: PS=Propensity score, aSD=absolute standardized difference, SD=Standard deviation, CCI=Charlson comorbidity index
