# Supplementary material for: COVID-19 infection and severe clinical outcomes in patients with kidney disease by vaccination status: a nationwide cohort study in Korea
Source: Epidemiol Health. 2024 Jul 17;46:e2024065. doi: 10.4178/epih.e2024065 (PMC11576527; doi:10.4178/epih.e2024065)
Supplement: Supplementary Material 4. — Time trend of incidence proportions of COVID-19 infection in unvaccinated cohort [file epih-46-e2024065-Supplementary-4.docx]

**Supplementary** **Material 4. Time trend of incidence proportions of COVID-19 infection in unvaccinated cohort**

**
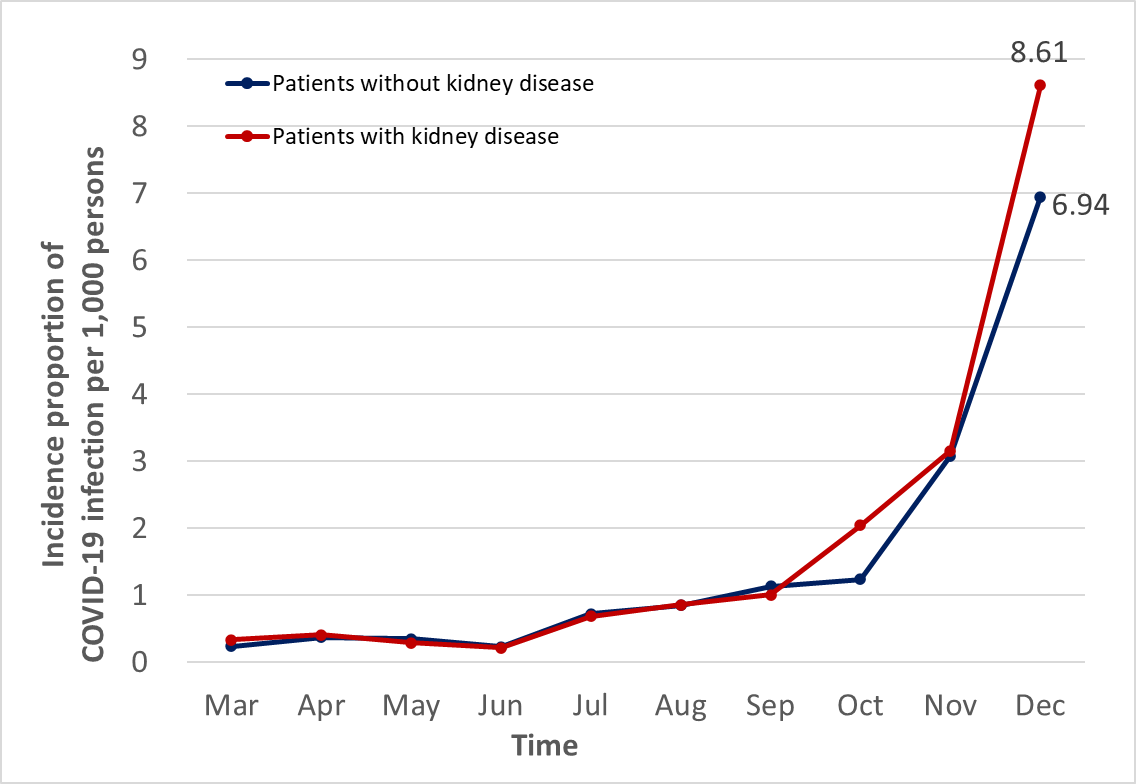
**
