# Supplementary material for: COVID-19 infection and severe clinical outcomes in patients with kidney disease by vaccination status: a nationwide cohort study in Korea
Source: Epidemiol Health. 2024 Jul 17;46:e2024065. doi: 10.4178/epih.e2024065 (PMC11576527; doi:10.4178/epih.e2024065)
Supplement: Supplementary Material 5. — Subgroup analysis on the risk of COVID-19 and severe clinical outcomes in patients with kidney disease compared to patients without kidney disease in vaccinated cohort [file epih-46-e2024065-Supplementary-5.docx]

**Supplementary Material 5. Subgroup analysis on the risk of COVID-19 and severe clinical outcomes in patients with kidney disease compared to patients without kidney disease in vaccinated cohort**

|  | | | **Patients with kidney disease** | | **Patients without kidney disease** | | **Adjusted HR** |
| --- | --- | --- | --- | --- | --- | --- | --- |
|  |  |  | **No. of events/total patients** | **IR per 1,000 pys** | **No. of events/total patients** | **IR per 1,000 pys** | **(95% CI)** |
| **COVID-19 infection** | | | | |  |  |  |
|  | **Age** | | | | | | |
|  |  | 12-17^a)^ | 1 / 241 | 27.1 | 2 / 964 | 12.9 | 6.20 (0.30-129.05) |
|  |  | 18-44^b)^ | 65 / 12,871 | 13.1 | 285 / 51,484 | 14.4 | 0.90 (0.69-1.18) |
|  |  | 45-64 | 398 / 57,455 | 14.8 | 1,464 / 229,820 | 13.6 | 1.08 (0.97-1.20) |
|  |  | ≥65 | 730 / 85,753 | 14.5 | 2,633 / 343,012 | 13.0 | 1.13 (1.04-1.23) |
|  | **Sex** | | | | | | |
|  |  | Male^c)^ | 687 / 88,844 | 15.0 | 2,654 / 355,376 | 14.4 | 1.04 (0.95-1.13) |
|  |  | Female | 510 / 67,878 | 14.0 | 1,864 / 271,512 | 12.7 | 1.12 (1.01-1.23) |
|  | **CCI** | | | | | | |
|  |  | 0^d)^ | 190 / 27,779 | 13.8 | 692 / 111,116 | 12.8 | 1.07 (0.91-1.26) |
|  |  | 1 | 315 / 42,662 | 14.5 | 1,179 / 170,648 | 13.4 | 1.09 (0.96-1.23) |
|  |  | 2^e)^ | 110 / 11,133 | 18.4 | 319 / 44,532 | 13.4 | 1.37 (1.11-1.71) |
|  |  | ≥3^f)^ | 181 / 20,645 | 16.3 | 607 / 82,580 | 13.6 | 1.21 (1.02-1.42) |
|  | **Vaccination dose** | | | |  |  |  |
|  |  | Partial | 120 / 155,089 | 5.4 | 481 / 620,356 | 5.4 | 1.03 (0.85-1.26) |
|  |  | Full | 1,061 / 155,572 | 19.6 | 3,978 / 622,288 | 18.2 | 1.10 (1.02-1.18) |
| **All-cause mortality** | | | | |  |  |  |
|  | **Age** | | | | | | |
|  |  | 12-17^a)^ | 0 / 241 | 0.0 | 0 / 964 | 0.0 | **-** |
|  |  | 18-44^b)^ | 0 / 12,871 | 0.0 | 0 / 51,484 | 0.0 | **-** |
|  |  | 45-64 | 2 / 57,455 | 0.1 | 4 / 229,820 | 0.0 | 2.00 (0.37-10.91) |
|  |  | ≥65 | 57 / 85,753 | 1.1 | 105 / 343,012 | 0.5 | 2.24 (1.62-3.09) |
|  | **Sex** | | | | | | |
|  |  | Male^c)^ | 29 / 88,844 | 0.6 | 49 / 355,376 | 0.3 | 2.45 (1.55-3.89) |
|  |  | Female | 27 / 67,878 | 0.7 | 56 / 271,512 | 0.4 | 1.97 (1.25-3.12) |
|  | **CCI** | | | | | | |
|  |  | 0^d)^ | 4 / 27,779 | 0.3 | 7 / 111,116 | 0.1 | 2.12 (0.62-7.25) |
|  |  | 1 | 9 / 42,662 | 0.4 | 15 / 170,648 | 0.2 | 2.45 (1.07-5.60) |
|  |  | 2^e)^ | 9 / 11,133 | 1.5 | 7 / 44,532 | 0.3 | 5.01 (1.86-13.45) |
|  |  | ≥3^f)^ | 20 / 20,645 | 1.8 | 21 / 82,580 | 0.5 | 3.74 (2.03-6.90) |
|  | **Vaccination dose** | | | |  |  |  |
|  |  | Partial | 7 / 155,089 | 0.3 | 15 / 620,356 | 0.2 | 2.20 (0.89-5.42) |
|  |  | Full | 48 / 155,572 | 0.9 | 100 / 622,288 | 0.5 | 1.99 (1.41-2.81) |
| **Hospitalization** | | | | |  |  |  |
|  | **Age** | | | | | | |
|  |  | 12-17^a)^ | 0 / 241 | 0.0 | 1 / 964 | 6.5 | **-** |
|  |  | 18-44^b)^ | 17 / 12,871 | 3.4 | 97 / 51,484 | 4.9 | 0.70 (0.42-1.18) |
|  |  | 45-64 | 186 / 57,455 | 6.9 | 659 / 229,820 | 6.1 | 1.12 (0.95-1.32) |
|  |  | ≥65 | 375 / 85,753 | 7.5 | 1,223 / 343,012 | 6.0 | 1.25 (1.11-1.40) |
|  | **Sex** | | | | | | |
|  |  | Male^c)^ | 339 / 88,844 | 7.4 | 1,198 / 355,376 | 6.5 | 1.13 (1.005-1.28) |
|  |  | Female | 238 / 67,878 | 6.5 | 829 / 271,512 | 5.6 | 1.17 (1.02-1.35) |
|  | **CCI** | | | | | | |
|  |  | 0^d)^ | 81 / 27,779 | 5.9 | 288 / 111,116 | 5.3 | 1.09 (0.85-1.39) |
|  |  | 1 | 150 / 42,662 | 6.9 | 507 / 170,648 | 5.8 | 1.20 (1.004-1.45) |
|  |  | 2^e)^ | 59 / 11,133 | 9.9 | 158 / 44,532 | 6.6 | 1.48 (1.10-1.99) |
|  |  | ≥3^f)^ | 95 / 20,645 | 8.6 | 289 / 82,580 | 6.5 | 1.33 (1.05-1.67) |
|  | **Vaccination dose** | | | |  |  |  |
|  |  | Partial | 65 / 155,089 | 2.9 | 276 / 620,356 | 3.1 | 0.97 (0.74-1.28) |
|  |  | Full | 523 / 155,572 | 9.6 | 1,853 / 622,288 | 8.5 | 1.19 (1.07-1.32) |
| **Emergency room visits** | | | | |  |  |  |
|  | **Age** | | | | | | |
|  |  | 12-17^a)^ | 0 / 241 | 0.0 | 0 / 964 | 0.0 | **-** |
|  |  | 18-44^b)^ | 7 / 12,871 | 1.4 | 27 / 51,484 | 1.4 | 1.05 (0.45-2.44) |
|  |  | 45-64 | 44 / 57,455 | 1.6 | 144 / 229,820 | 1.3 | 1.21 (0.86-1.69) |
|  |  | ≥65 | 73 / 85,753 | 1.5 | 230 / 343,012 | 1.1 | 1.29 (0.99-1.68) |
|  | **Sex** | | | | | | |
|  |  | Male^c)^ | 65 / 88,844 | 1.4 | 228 / 355,376 | 1.2 | 1.14 (0.87-1.51) |
|  |  | Female | 55 / 67,878 | 1.5 | 181 / 271,512 | 1.2 | 1.25 (0.92-1.68) |
|  | **CCI** | | | | | | |
|  |  | 0^d)^ | 22 / 27,779 | 1.6 | 56 / 111,116 | 1.0 | 1.51 (0.92-2.48) |
|  |  | 1 | 30 / 42,662 | 1.4 | 110 / 170,648 | 1.2 | 1.11 (0.74-1.66) |
|  |  | 2^e)^ | 10 / 11,133 | 1.7 | 21 / 44,532 | 0.9 | 1.89 (0.89-4.01) |
|  |  | ≥3^f)^ | 24 / 20,645 | 2.2 | 60 / 82,580 | 1.3 | 1.64 (1.02-2.64) |
|  | **Vaccination dose** | | | |  |  |  |
|  |  | Partial | 12 / 155,089 | 0.5 | 45 / 620,356 | 0.5 | 1.12 (0.59-2.11) |
|  |  | Full | 108 / 155,572 | 2.0 | 371 / 622,288 | 1.7 | 1.19 (0.95-1.49) |

**Abbreviation**: IR=Incidence rate, pys=person-years, HR=Hazard ratio, CI=Confidence interval, CCI=Charlson comorbidity index

^a)^ Covariates with imbalance after propensity score matching (age, CCI, anemia, cancer, congestive heart failure, depression, diabetes, liver disease) were adjusted.

^b)^ Covariates with imbalance after propensity score matching (cancer, chronic lung disease, hyperlipidemia, liver disease, second vaccine type) were adjusted.

^c)^ Covariates with imbalance after propensity score matching (chronic lung disease) were adjusted.

^d)^ Covariates with imbalance after propensity score matching (liver disease, first vaccine type, second vaccine type) were adjusted.

^e)^ Covariates with imbalance after propensity score matching (first vaccine type) were adjusted.

^f)^ Covariates with imbalance after propensity score matching (diabetes) were adjusted.
