# Supplementary material for: COVID-19 infection and severe clinical outcomes in patients with kidney disease by vaccination status: a nationwide cohort study in Korea
Source: Epidemiol Health. 2024 Jul 17;46:e2024065. doi: 10.4178/epih.e2024065 (PMC11576527; doi:10.4178/epih.e2024065)
Supplement: Supplementary Material 6. — Subgroup analysis on the risk of COVID-19 and severe clinical outcomes in patients with kidney disease compared to patients without kidney disease in unvaccinated cohort [file epih-46-e2024065-Supplementary-6.docx]

**Supplementary Material 6. Subgroup analysis on the risk of COVID-19 and severe clinical outcomes in patients with kidney disease compared to patients without kidney disease in unvaccinated cohort**

|  | | | **Patients with kidney disease** | | **Patients without kidney disease** | | **Adjusted HR** |
| --- | --- | --- | --- | --- | --- | --- | --- |
|  |  |  | **No. of events/total patients** | **IR per 1,000 pys** | **No. of events/total patients** | **IR per 1,000 pys** | **(95% CI)** |
| **COVID-19 infection** | | | | |  |  |  |
|  | **Age** | | | | | | |
|  |  | 12-17^a)^ | 7 / 462 | 19.7 | 20 / 1,848 | 14.2 | 1.36 (0.57-3.24) |
|  |  | 18-44^b)^ | 84 / 13,428 | 12.2 | 262 / 53,712 | 9.3 | 1.26 (0.98-1.61) |
|  |  | 45-64^c)^ | 183 / 58,828 | 7.6 | 754 / 235,312 | 7.7 | 0.95 (0.80-1.11) |
|  |  | ≥65 | 245 / 91,987 | 8.8 | 750 / 367,948 | 6.9 | 1.19 (1.03-1.37) |
|  | **Sex** | | | | | | |
|  |  | Male^d)^ | 289 / 93,766 | 8.5 | 1,003 / 375,064 | 7.4 | 1.10 (0.97-1.26) |
|  |  | Female^e)^ | 237 / 72,648 | 9.1 | 751 / 290,592 | 7.5 | 1.11 (0.96-1.29) |
|  | **CCI** | | | | | | |
|  |  | 0^f)^ | 81 / 27,237 | 7.6 | 311 / 108,948 | 7.6 | 0.94 (0.74-1.20) |
|  |  | 1 | 162 / 48,866 | 9.0 | 541 / 195,464 | 7.6 | 1.08 (0.91-1.29) |
|  |  | 2 | 30 / 10,980 | 7.9 | 116 / 43,920 | 7.7 | 0.98 (0.66-1.46) |
|  |  | ≥3^g)^ | 83 / 22,108 | 10.7 | 246 / 88,432 | 8.1 | 1.22 (0.95-1.57) |
| **All-cause mortality** | | | | |  |  |  |
|  | **Age** | | | | | | |
|  |  | 12-17^a)^ | 0 / 462 | - | 0 / 1,848 | - | **-** |
|  |  | 18-44^b)^ | 0 / 13,428 | - | 0 / 53,712 | - | **-** |
|  |  | 45-64^c)^ | 3 / 58,828 | 0.1 | 5 / 235,312 | 0.1 | 2.11 (0.50-8.83) |
|  |  | ≥65 | 47 / 91,987 | 1.7 | 88 / 367,948 | 0.8 | 1.85 (1.30-2.64) |
|  | **Sex** | | | | | | |
|  |  | Male^d)^ | 29 / 93,766 | 0.9 | 50 / 375,064 | 0.4 | 2.18 (1.38-3.45) |
|  |  | Female^e)^ | 26 / 72,648 | 1.0 | 41 / 290,592 | 0.4 | 2.09 (1.28-3.42) |
|  | **CCI** | | | | | | |
|  |  | 0^f)^ | 1 / 27,237 | 0.1 | 8 / 108,948 | 0.2 | 0.36 (0.05-2.87) |
|  |  | 1 | 8 / 48,866 | 0.4 | 14 / 195,464 | 0.2 | 1.91 (0.80-4.55) |
|  |  | 2 | 3 / 10,980 | 0.8 | 11 / 43,920 | 0.7 | 1.00 (0.28-3.57) |
|  |  | ≥3^g)^ | 15 / 22,108 | 1.9 | 26 / 88,432 | 0.9 | 1.96 (1.03-3.70) |

| **Hospitalization** | | | | |  |  |  |
| --- | --- | --- | --- | --- | --- | --- | --- |
|  | **Age** | | | | | | |
|  |  | 12-17^a)^ | 0 / 462 | - | 8 / 1,848 | 5.7 | **-** |
|  |  | 18-44^b)^ | 52 / 13,428 | 7.5 | 152 / 53,712 | 5.4 | 1.36 (0.99-1.87) |
|  |  | 45-64^c)^ | 82 / 58,828 | 3.4 | 413 / 235,312 | 4.2 | 0.77 (0.61-0.98) |
|  |  | ≥65 | 111 / 91,987 | 4.0 | 361 / 367,948 | 3.3 | 1.10 (0.89-1.36) |
|  | **Sex** | | | | | | |
|  |  | Male^d)^ | 134 / 93,766 | 4.0 | 516 / 375,064 | 3.8 | 0.99 (0.81-1.19) |
|  |  | Female^e)^ | 118 / 72,648 | 4.5 | 374 / 290,592 | 3.7 | 1.10 (0.89-1.35) |
|  | **CCI** | | | | | | |
|  |  | 0^f)^ | 38 / 27,237 | 3.6 | 168 / 108,948 | 4.1 | 0.81 (0.57-1.16) |
|  |  | 1 | 67 / 48,866 | 3.7 | 277 / 195,464 | 3.9 | 0.87 (0.67-1.14) |
|  |  | 2 | 16 / 10,980 | 4.2 | 61 / 43,920 | 4.1 | 1.00 (0.57-1.73) |
|  |  | ≥3^g)^ | 42 / 22,108 | 5.4 | 120 / 88,432 | 3.9 | 1.26 (0.89-1.80) |
| **Emergency room visits** | | | | |  |  |  |
|  | **Age** | | | | | | |
|  |  | 12-17^a)^ | 0 / 462 | - | 3 / 1,848 | 2.1 | **-** |
|  |  | 18-44^b)^ | 18 / 13,428 | 2.6 | 26 / 53,712 | 0.9 | 2.70 (1.47-4.93) |
|  |  | 45-64^c)^ | 17 / 58,828 | 0.7 | 79 / 235,312 | 0.8 | 0.83 (0.49-1.39) |
|  |  | ≥65 | 32 / 91,987 | 1.1 | 80 / 367,948 | 0.7 | 1.42 (0.94-2.14) |
|  | **Sex** | | | | | | |
|  |  | Male^d)^ | 43 / 93,766 | 1.3 | 122 / 375,064 | 0.9 | 1.34 (0.94-1.89) |
|  |  | Female^e)^ | 26 / 72,648 | 1.0 | 80 / 290,592 | 0.8 | 1.13 (0.73-1.76) |
|  | **CCI** | | | | | | |
|  |  | 0^f)^ | 15 / 27,237 | 1.4 | 36 / 108,948 | 0.9 | 1.47 (0.80-2.70) |
|  |  | 1 | 16 / 48,866 | 0.9 | 65 / 195,464 | 0.9 | 0.87 (0.50-1.51) |
|  |  | 2 | 2 / 10,980 | 0.5 | 14 / 43,920 | 0.9 | 0.54 (0.12-2.37) |
|  |  | ≥3^g)^ | 8 / 22,108 | 1.0 | 19 / 88,432 | 0.6 | 1.65 (0.72-3.79) |

**Abbreviation**: IR=Incidence rate, pys=person-years, HR=Hazard ratio, CI=Confidence interval, CCI=Charlson comorbidity index

^a)^ Covariates with imbalance after propensity score matching (anemia, cardiac dysrhythmias, chronic lung disease, hyperlipidemia, hypothyroidism, liver disease) were adjusted.

^b)^ Covariates with imbalance after propensity score matching (hyperlipidemia, liver disease) were adjusted.

^c)^ Covariates with imbalance after propensity score matching (chronic lung disease) were adjusted.

^d)^ Covariates with imbalance after propensity score matching (chronic lung disease) were adjusted.

^e)^ Covariates with imbalance after propensity score matching (chronic lung disease) were adjusted.

^f)^ Covariates with imbalance after propensity score matching (chronic lung disease) were adjusted.

^g)^ Covariates with imbalance after propensity score matching (diabetes) were adjusted.
